# Childhood Neglect rather than Abuse Is More Strongly Associated with Anhedonia across Major Depression and Obsessive-Compulsive Disorder Patients and University Students

**DOI:** 10.1155/2023/2429889

**Published:** 2023-10-20

**Authors:** Jie Fan, Yan Han, Jie Xia, Xiang Wang, Qian Liu, Yao Liu, Jingjie Lu, Quanhao Yu, Yanjie Yang, Xiongzhao Zhu

**Affiliations:** ^1^Medical Psychological Center, The Second Xiangya Hospital, Central South University, Changsha, Hunan 410011, China; ^2^Medical Psychological Institute of Central South University, Changsha, Hunan 410011, China; ^3^National Clinical Research Center for Mental Disorders, Changsha 410011, Hunan, China; ^4^Department of Psychiatry and National Clinical Research Center for Mental Disorders, The Second Xiangya Hospital, Central South University, Changsha, Hunan 410011, China; ^5^Medical Psychology Department, Harbin Medical University, Harbin 150081, China

## Abstract

**Background:**

According to the dimensional model of adversity, the deprivation and threat dimensions of CT influence distinct neural circuits and have different developmental outcomes. The present study compared neglect and abuse subtypes which are representative of deprivation and threat dimensions of CT prediction of anhedonia in MDD and OCD patients and university students.

**Methods:**

A total of 305 patients with MDD, 152 patients with OCD, and 2110 university students fulfilled the Childhood Trauma Questionnaire to identify neglect and abuse subtypes of CT. Different aspects of anhedonia were measured. Hierarchical linear regression analysis was conducted to identify subtypes of trauma as predictors of different aspects of anhedonia in MDD and OCD patients and university students, respectively.

**Results:**

Childhood neglect, not abuse, showed association with anticipatory and state anhedonia in OCD patients and anticipatory, consummatory, physical, and state anhedonia in MDD patients and university students. Both childhood neglect and abuse were associated with social anhedonia in university students, but the neglect type showed greater magnitude. In terms of more specific categories, emotional neglect was the type of CT demonstrating strongest magnitude of association with most of the anhedonia aspects.

**Conclusions:**

Findings revealed that deprivation, rather than threat, was the more influential adversity dimension for the individuals' anhedonia presentations.

## 1. Introduction

Childhood trauma (CT) comprises any behavior by parents or other caregivers that induces essential, potential, or threatening harm to a child, even if not intentionally [1]. It exerts a lasting impact on cognition, emotion, and behavior in adulthood and is well recognized as a major risk factor for psychopathology [2, 3]. However, the mechanism of how CT is linked to mental health problems remains unclear. Exploring the behavioral outcome affected by CT might provide a key insight into solving this issue.

Anhedonia, defined as the reduced ability to experience pleasure or a loss of desire to engage in enjoyable activities [4], was proposed as a potential behavioral outcome associated with CT [5, 6]. Anhedonia is presented in various psychiatric disorders including major depression disorder (MDD), bipolar disorder, obsessive-compulsive disorder (OCD), substance abuse, and schizophrenia, representing a dimension of psychopathology that crosses diagnostic boundaries [7, 8]. Anhedonia is not only a symptom correlate but also a risk marker in psychopathology. A higher level of anhedonia was found in individuals who experienced childhood adversity [6, 9, 10], and it was suggested to be a predictor of future mental illness [11, 12]. To conclude, these findings indicate that anhedonia might be a potential vulnerability in the development of psychopathology associated with CT.

Yet, despite that the existing preliminary research has explored the relationship between CT and anhedonia, several questions remained unanswered. First, the effects of different CT dimensions on anhedonia have not been clarified. According to the recently proposed dimensional model of adversity, CT can be distinguished into deprivation and threat dimensions [13]. Threat refers to the experience involving actual harm or the perception of potential harm, with abuse being the prime example. Unlike threat, deprivation is a dimension of experience characterized by the absence of expected cognitive inputs, social stimulation, and consistent interactions with adults, in which neglect is the most representative type. Previous findings have suggested that these two CT dimensions were associated with distinct neural circuits and therefore contribute to the development of psychopathology through different mechanisms [13, 14]. Specifically, it was revealed that individuals exposed to abuse may develop disruptions mainly in fear-learning processing [13, 15]. Compared to abuse, children who were exposed to neglect exhibited deficits more frequently in cognitive dysfunction and reward-learning processing [16–19]. This may be attributed to the fact that children raised in the absence of stable caregiving may experience significant reductions in receiving feedback, which is essential for brain and cognition development. Notably, it was well established that deficits in reward processing are a central mechanism underlying anhedonia [20], which raises the possibility that CT dimension of deprivation is more strongly associated with anhedonia than threat dimension. However, this hypothesis needs to be further examined as previous studies have not directly compared the effects of CT dimensions on anhedonia.

Another important question is whether CT has differential effects on various aspects of anhedonia. It is important to note that anhedonia is not a unitary concept but can be parsed into different aspects according to different criteria. For example, according to the cognitive phase of the rewarding process, anhedonia can be divided into anticipatory and consummatory aspects [21]. Based on the nature of the pleasurable stimulus, there are physical and social aspects of anhedonia [22]. In addition, anhedonia can be experienced as either a state characteristic, such as transient anhedonic emotions, or an enduring anhedonic mood or trait that persists over time [23]. Different aspects of anhedonia may exhibit overlapping neural substrates, such as the involvement of the reward system, but they can also have distinct neural correlates [24]. It remains to be explored whether different dimensions of CT have a general effect on anhedonia or if their impact is limited to specific aspects.

Lastly, as anhedonia can be observed among different populations, whether the predictive association between neglect and abuse subtypes of CT and anhedonia could be replicated in independent populations should be confirmed. Among various psychiatric disorders, anhedonia was found to be one of the core symptoms of MDD and an important feature of OCD. In MDD, deficits in almost all aspects of pleasure were observed [25]. In OCD, a previous study has revealed that 28.3% of patients with OCD demonstrated clinically significant anhedonia symptoms, and the anhedonia in OCD was independent of their depressive symptoms [8, 26]. Besides the prevalence of anhedonia in both MDD and OCD, CT was also found to be a common risk for MDD and OCD [27]. Based on these considerations, to further confirm the relationship between CT dimension and anhedonia, we have also included individuals with MDD and OCD as our clinical samples in addition to university students.

To sum up, this present study is aimed at distinguishing the effect of neglect and abuse CT subtypes on different dimensions of anhedonia. Specifically, we investigated the relationship between CT subtypes and anhedonia in patients with MDD and OCD and university students. We hypothesized that childhood neglect rather than abuse is more strongly associated with anhedonia across samples.

## 2. Methods

### 2.1. Participants

A total of 305 MDD patients, 152 OCD patients, and 2110 university students were included in the present study. Patients were recruited from the psychology clinic at the Second Xiangya Hospital, Changsha, China. Two experienced psychiatrists confirmed the diagnosis of MDD or OCD and comorbidity for each patient according to the Structured Clinical Interview for the DSM-IV Axis I (SCID-I). Participants were excluded if they had a lifetime history of significant head trauma or met the criteria for axis I psychiatric disorder comorbidity. Eighty-nine MDD patients (29.2%) and 53 OCD patients (34.9%) were taking medications when recruited.

The university students were enrolled in the year of 2018. They came from 4 universities in two provinces of China. We provided survey information on the questionnaires and distributed them to students during a class break. A total of 2220 students were initially tested, and participants with missing values were subsequently excluded. This process led to a final sample of 2110 participants who provided fully completed questionnaires, which yielded a response rate of 95%.

The study was approved by the Ethics Committee of the Second Xiangya Hospital of Central South University, and all the participants provided written informed consent.

### 2.2. Clinical and Psychological Assessments

Childhood experiences of adversity were evaluated using the Childhood Trauma Questionnaire (CTQ) [28]. The CTQ is a widely used retrospective self-report tool which assesses five specific CT forms: neglect (including emotional and physical neglect) and abuse (including emotional, physical, and sexual abuse). The total scores of the neglect and abuse forms were calculated, respectively, to create the deprivation and threat exposure composites. Dichotomous variables of exposure for each CTQ subscale were created using cut-off scores for moderate-to-severe CT as outlined in the CTQ manual: physical neglect ≥ 10, physical abuse ≥ 10, emotional neglect ≥ 15, emotional abuse ≥ 13, and sexual abuse ≥ 8 [29].

Several assessments were adopted to measure the different aspects of anhedonia. Specifically, the Temporal Experience of Pleasure Scale (TEPS) was administrated to capture the anticipatory and consummatory aspects of anhedonia [21]. TEPS consists of two subscales assessing anticipatory (TEPS_ANT) and consummatory pleasure (TEPS_CON). A lower score represents a more severe anhedonia. The revised Chapman Physical Anhedonia Scale (PAS) and Social Anhedonia Scale (SAS) were administrated to evaluate physical and social anhedonia, respectively [30, 31]. The cut-off scores for “with anhedonia” of PAS and SAS for male and female were 28 and 20 and 20 and 16, respectively [22, 30]. PAS, SAS, and TEPS all focus on lifetime hedonic responses, making them all trait-like measures. We also administrated the Snaith-Hamilton Pleasure Scale (SHAPS) to evaluate the state anhedonia [32]. SHAPS is a 14-item, self-report scale which is designed to assess an individual' hedonic experience in the most recent few days. It was rated on a 4-point Likert scale ranging from 1 (strongly disagree) to 4 (strongly agree). According to original scale [32], the cut-off score of SHAPS is 2 in the case that either of the “disagree” options scores 1 and either of the “agree” options scores 0. It should be noted that we did not include SHAPS in the initial recruitment of MDD and OCD patients; thus, only a certain part of the participants (103 MDD patients and 67 OCD patients) have the records for this particular scale.

Participants also finished the Beck Depression Inventory (BDI) [33], the State-Trait Anxiety Inventory (STAI) [34], and the Perceived Stress Scale (PSS) [35] to evaluate depression, anxiety, and recent perceived stress level. Additionally, patients with OCD completed the Yale-Brown Obsessive Compulsive Scale (Y-BOCS) to evaluate symptom severity [36].

### 2.3. Statistical Analysis

The *t*-tests and chi-square tests were used to compare the differences of anhedonia among different groups. Hierarchical regressions were carried out to investigate the associations between CT subtypes and different anhedonia aspects in MDD and OCD patients and university students, respectively. In the regression models, age, gender, years of education, and PSS and STAI scores were entered as predictors/covariates in step 1. For the OCD group, covariates in step 1 additionally included YBOCS scores. The correlation analyses between CT subtypes and different anhedonia aspects were firstly run to screen the predictors in step 2: only the CT subtypes showing significant correlations with anhedonia were included. In step 2, regression models including neglect and/or abuse scores as predictor were conducted to distinguish the effect of neglect and abuse subtypes of CT on anhedonia. To determine the most influential specific CT form on anhedonia, regression models with 5 specific neglect and/or abuse forms included as predictors in step 2 were also carried out using the stepwise method. Variance inflation factor (VIF) was adopted to evaluate collinearity among predictors in regression models. Corrected *p* values (0.05 divided by the number of CT predictors in step 2, Bonferroni correction) were considered as significance thresholds to reduce the risk of type I errors due to multiple testing. All data were analyzed using SPSS v21.0 IBM.

## 3. Results

### 3.1. Demographical Variables and CT and Anhedonia Prevalence

Demographics and trauma frequencies of MDD patients, OCD patients, and university students are presented in Table 1. Prevalence and group differences of anhedonia are summarized in Table 2. The mean age of the three samples was 22.45 (SD = 5.64), 22.63 (SD = 5.42), and 19.79 (SD = 1.26), respectively; 69.2% of the MDD patients, 45.4% of the OCD patients, and 53% of the university students were females. Patients with MDD scored highest on depression and anxiety assessments (Table 1).

Approximately 69.8% of the MDD patients, 65.8% of the OCD patients, and 33.6% of the university students reported trauma experience in childhood. Neglect was reported by 62.6% of the MDD group, 55.3% of the OCD group, and 30.0% of the university students; abuse was reported by 38.7% of the MDD group, 32.9% of the OCD group, and 9.00% of university students. The two most frequently reported CT forms in all three samples were emotional neglect (55.4%, 34.9%, and 11.1% in MDD patients, OCD patients, and university students, respectively) and physical neglect (39.0%, 40.8%, and 25.8%). About 25% of the MDD patients, 13.8% of the OCD patients, and 3.0% of the university students had reported 3 or more types of CT (Table 1).

Of the 2110 university students, 19.8% presented state anhedonia, 17.4% presented physical trait anhedonia, and 15.3% presented social trait anhedonia. The percentages for MDD were 82.5%, 72.5%, and 74.1% and for OCD were 65.7%, 48.7%, and 47.4%. Patients with MDD had the most severe anhedonia, followed by patients with OCD. University students with CT had more severe anhedonia on all 5 anhedonia aspects than students without CT. The percentages of individuals who presented state and social trait anhedonia in MDD patients with CT were higher than those without CT. No significant group differences on anhedonia assessments between OCD with and without CT were detected (Table 2).

### 3.2. Regression of Different Aspects of Anhedonia on CT Subtypes

Table 3 presents the correlations between CT subtypes and anhedonia aspects.  Table 4 presents the regression of neglect and/or abuse on anhedonia for the MDD group, OCD group, and university students. Tables 5 and 6 illustrate the regression of five specific neglect and abuse forms on anhedonia for the MDD group, OCD group, and university students, respectively.

In patients with MDD, the neglect total score and physical and emotional neglect subscale scores were correlated with all five anhedonia aspects, with the correlation coefficients ranging from 0.13 to 0.26 (*ps* < 0.05) (Table 3). Abuse total score and emotional abuse subscale score were significantly correlated with SAS (*ps* < 0.01). Regression analysis revealed that, after controlling for age, gender, years of education, anxiety level, and recent stress level, only neglect showed significant association with all five anhedonia aspects (TEPS_ANT: *β* = −0.14, *p* = 0.020; TEPS_CON: *β* = −0.14, *p* = 0.015; PAS: *β* = 0.19, *p* < 0.001; SAS: *β* = 0.18, *p* = 0.002; and SHAPS: *β* = 0.23, *p* = 0.015). More specifically, emotional neglect showed the strongest association with anticipatory (*β* = −0.14, *p* = 0.020), consummatory (*β* = −0.14, *p* = 0.015), physical (*β* = 0.19, *p* < 0.001), and state anhedonia (*β* = 0.23, *p* = 0.015), while physical neglect showed the greatest magnitude of association with social anhedonia (*β* = 0.18, *p* = 0.002) (Tables 4 and 5).

In the OCD group, significant correlations were observed between TEPS_ANT and neglect total score and emotional neglect subscale score and between SHAPS and neglect total score and physical and emotional neglect scores (*ps* < 0.05) (Table 3). Regression analysis revealed that, after controlling for age, gender, years of education, anxiety level, recent stress level, and OCD symptom severity, neglect showed significant association with anticipatory (*β* = −0.21, *p* = 0.015) and state anhedonia (*β* = 0.26, *p* = 0.020). Specifically, emotional neglect showed the strongest association with anticipatory anhedonia (*β* = −0.22, *p* = 0.008), and physical neglect showed the strongest association with state anhedonia (*β* = 0.27, *p* = 0.018) (Tables 4 and 5).

Regarding the university students, both neglect and abuse were correlated with the five anhedonia aspects (*ps* < 0.01) (Table 3). Regression analysis revealed that, after controlling for age, gender, years of education, anxiety level, and recent stress level, only neglect showed significant association with the anticipatory (*β* = −0.16, *p* < 0.001), consummatory (*β* = −0.12, *p* < 0.001), physical (*β* = 0.10, *p* < 0.001), and state anhedonia (*β* = 0.14, *p* < 0.001). Both neglect and abuse showed significant associations with social anhedonia, while neglect (*β* = 0.11, *p* < 0.001) showed more significant effect compared to the abuse (*β* = 0.08, *p* < 0.001). Among the specific forms of CT, emotional neglect showed the strongest association with all five aspects of anhedonia (TEPS_ANT: *β* = −0.17, *p* < 0.001; TEPS_CON: *β* = −0.10, *p* < 0.001; PAS: *β* = 0.10, *p* < 0.001; SAS: *β* = 0.13, *p* < 0.001; and SHAPS: *β* = 0.14, *p* < 0.001) (Tables 4 and 6).

The association between CT subtypes and anhedonia revealed by the regression analyses all remained significant after the Bonferroni correction. For all the regression models, the VIF for all explanatory variables was less than 5, indicating that multicollinearity should not be a problem in the models. Detailed results for the prediction of covariates in step 1 for all regression models are presented in supplementary tables (Table S1-S3).

## 4. Discussion

The present study examined the different effects of childhood neglect and abuse on anhedonia in 3 different samples. We found that in patients with MDD, neglect was more associated with all five anhedonia aspects compared to abuse. In patients with OCD, neglect, not abuse, was associated with anticipatory and state anhedonia. Among university students, neglect, rather than abuse, showed association with anticipatory, consummatory, physical, and state anhedonia. Both neglect and abuse were associated with social anhedonia, but neglect had a greater influence. Regarding the more specific forms of CT, emotional neglect had the strongest association with most aspects of anhedonia. This consistent pattern of relationship between neglect CT subtypes and anhedonia across different samples suggested that deprivation dimension of CT may be a primary factor associated with anhedonia.

The prevalence of trauma during childhood in university students was 33.6% in the current study, which was consistent with previous WHO surveys [27]. In line with previous findings [1], neglect was a more prevalent dimension of CT among patients with MDD and OCD and healthy individuals. In addition, previous studies also observed severer and more prevalent CT in MDD and OCD patients, which was consistent with our results [37, 38]. Individuals with MDD scored highest in the anhedonia assessments, which was in accordance with the clinical features. Around 15.3-19.8% of university students reported at least one type of anhedonia, and a higher percentage of students were exposed to childhood adversity, which highlighted the need for increased attention to the potential risks associated with childhood adversity on mental health outcomes.

Regarding the relationship between CT and anhedonia, the most important finding of the present study was that anhedonia was more strongly associated with neglect than abuse across MDD and OCD patients and university students. Anhedonia manifests as consistent and marked decrease in interest or pleasure in daily activities. Dysfunction in the brain's reward system, centered by the frontal-striatal circuit, was the main underlying mechanism of different aspects of anhedonia [24]. Deprivation is the core feature of neglect [39]. Researchers have proposed that neglect and abuse might influence development through different underlying mechanisms, among which emotion learning, including fear, and reward learning are particularly important [15, 40]. Compared to abuse, which affects later development mainly through disruptions in fear learning, the atypical reward-learning processing might be particularly observed in individuals who experienced neglect. In this regard, previous behavioral studies have found that children exposed to early deprivation showed reduced learning on a probabilistic reward-learning task and revised monetary incentive delay (MID) task [18, 19]. On the level of brain activity, children exposed to early deprivation were found to have reduced ventral striatum activity to positive social images and reduced ventral striatum response during reward anticipatory [16, 17]. These findings together suggested that the dysfunction in reward system and the deficits in reward learning might explain the link between neglect and anhedonia. Childhood neglect may deprive children of numerous environmental experiences necessary for the normal development of the human brain. Children who have experienced neglect may receive less attention and care from caregivers, resulting in less frequency of receiving feedback related to rewards and thus affecting reward learning. In terms of the more specific neglect type, emotional neglect had the strongest association with most aspect of anhedonia, suggesting that more attention should be paid to the emotional needs of children to prevent the potential development of anhedonia later in life. The present finding extended the previous literature by revealing the pronounced effect of neglect rather than abuse on anhedonia [6, 9, 10]. Our findings were consistent with our recent study which was conducted in another independent sample and found that neglect was also associated with changes in anhedonia over time [41]. In addition, the present results were also in line with our prior animal studies [42] which identified different stress paradigms inducing dissimilar depressive behaviors in male rats. Specifically, it was found that the maternal deprivation would induce severe anhedonia, indicating that neglect might impair the ability to experience pleasure in adulthood.

Notably, we also detected the effect of abuse on social anhedonia in university students, although to a lesser extent compared to neglect. Social anhedonia refers to the decreased enjoyment and interest specifically in the social interactions. Unlike other aspects of anhedonia, social anhedonia was not only related to deficits in reward system underlying hedonic experience but also associated with social brain subserving the social cognition processes [43–45]. Social cognition includes processes such as the perception of social cues, experience sharing, mentalizing, and emotion experiencing and regulation [46]. Correspondingly, social brain includes large-scale brain regions underlying social cognition, with the amygdala as a hub [47]. Previous studies have found the effect of abuse on the structure and function of the amygdala, facial emotion processing, and neural correlates of emotion regulation [48–50]. These findings raise the possibility that abuse may not affect social anhedonia through reward system, but via the influence on the development of social brain and social cognition. Children who grew up in abusive environment may become sensitive to subtle social cues and have difficulty making social judgements, hence hindering their enthusiasm for social interaction and reducing their ability to feel interpersonal pleasure. It should be pointed out that neglect can also influence brain regions included in the social brain [39]. Hence, both the reward system and the social brain might be involved in the link between neglect and social anhedonia. Association between abuse and social anhedonia was only detected in university students in the present study. Moreover, although we detected similar pattern of association between neglect and anhedonia across three samples, the relationship between neglect and five different aspects of anhedonia was established in MDD patients and university students, while the association between state and anticipatory anhedonia and neglect was identified in OCD patients only. The potential interaction between disorder diagnosis and childhood adversity might account for, at least in part, the observed difference. However, this should be further investigated by the future studies.

Several limitations should be addressed when interpreting the results of the present study. First, CTQ is a retrospective self-report scale. Individuals might hold some inaccurate memory of maltreatment history which may induce assessment bias. Second, the association between CT and anhedonia revealed in the present study could not necessarily imply a causal relationship. The way neglect impacts the development trajectory of anhedonia should be better investigated by future longitudinal studies. Third, we established the association between childhood neglect and anhedonia in MDD and OCD patients and university students; though we tend to believe that this pattern of relationship can be replicated in other samples, it should be further confirmed.

## 5. Conclusion

In conclusion, this study found that among childhood adversity subtypes, neglect had a stronger association with anhedonia in adulthood compared to abuse. These findings highlight the role of neglect, a representative of CT dimension of deprivation, in influencing anhedonia, which may provide insights into the mechanisms by which CT leads to psychopathology and suggest the importance of early interventions for families in such circumstances.

## Figures and Tables

**Table 1:** Demographical and psychological variables in MDD and OCD patients and university students.

Variables	MDD (*N* = 305)	OCD (*N* = 152)	University students (*N* = 2110)
Age	16-49, 22.45 (5.64)	14-43, 22.63 (5.42)	17-28, 19.79 (1.26)
Gender (female, %)	211, 69.2%	69, 45.4%	1118, 53%
Education years	13.79 (2.47)	13.93 (2.56)	12.94 (1.01)
Medicated (%)	89, 29.2%	53, 34.9%	—
SAI	58.83 (10.47)	43.76 (6.55)	38.72 (9.91)
TAI	61.68 (7.76)	48.30 (6.08)	41.28 (9.37)
BDI	30.15 (9.48)	21.69 (10.08)	8.55 (6.96)
PSS	36.63 (5.54)	21.32 (4.20)	15.22 (5.90)
Y-BOCS	—	20.43 (6.22)	—
TEPS_ANT	35.49 (9.39)	41.34 (8.06)	44.43 (8.12)
TEPS_CON	30.51 (8.01)	32.94 (7.38)	37.10 (7.44)
PAS	30.00 (11.00)	25.40 (11.20)	16.12 (7.57)
PAS (yes, %)	221, 72.5%	74, 48.7%	367, 17.4%
SAS	21.29 (6.90)	18.20 (8.74)	11.08 (5.94)
SAS (yes, %)	226, 74.1%	72, 47.4%	322, 15.3%
SHAPS^a^	33.70 (5.73)	32.06 (5.61)	24.07 (5.91)
SHAPS^a^ (yes, %)	85, 82.5%	44, 65.7%	418, 19.8%
CTQ; with CT, %	48.07 (12.82); 213, 69.8%	43.61 (10.40); 100, 65.8%	35.02 (8.05); 709, 33.6%
Neglect; with neglect, %	24.79 (7.46); 191, 62.6%	21.88 (6.26); 84, 55.3%	17.48 (5.73); 632, 30.0%
Abuse; with abuse, %	23.28 (7.37); 118, 38.7%	21.73 (6.36); 50, 32.9%	17.54 (3.64); 190, 9.00%
Only neglect, %	95, 31.1%	50, 32.9%	519, 24.6%
Only abuse, %	22, 7.2%	16, 10.5%	77, 3.6%
Mixed	96, 31.5%	34, 22.4%	113, 5.4%
EN; with EN, %	14.89 (5.03); 169, 55.4%	12.80 (4.54); 53, 34.9%	9.55 (3.86); 235, 11.1%
PN; with PN, %	9.89 (3.35); 119, 39.0%	9.09 (2.82); 62, 40.8%	7.93 (2.79); 545, 25.8%
EA; with EA, %	10.09 (4.24); 79, 25.9%	9.03 (3.77); 28, 18.4%	6.52 (2.00); 47, 2.2%
PA; with PA, %	7.16 (3.16); 53, 17.4%	6.64 (2.57); 20, 13.1%	5.62 (1.58); 73, 3.5%
SA; with SA, %	6.02 (2.14); 46, 15.1%	6.06 (2.20); 20, 13.1%	5.40 (1.25); 108, 5.1%
CT_number			
1, %	69, 22.6%	46, 30.3%	502, 23.8%
2, %	68, 22.3%	33, 21.7%	141, 6.7%
3, %	45, 14.8%	17, 11.2%	43, 2.0%
4, %	25, 8.2%	4, 2.6%	20, 0.9%
5, %	6, 2.0%	0	3, 0.1%

^a^Sample sizes in MDD and OCD with SHAPS were 103 and 67. OCD: obsessive-compulsive disorder; MDD: major depression disorder; STAI: State-Trait Anxiety Inventory; BDI: Beck Depression Inventory; PSS: Perceived Stress Scale; Y-BOCS: Yale-Brown Obsessive Compulsive Scale; TEPS_ANT: anticipatory pleasure subscale of Temporal Experience of Pleasure Scale; TEPS_CON: consummatory pleasure subscale of TEPS; PAS: Physical Anhedonia Scale; SAS: Social Anhedonia Scale; SHAPS: Snaith-Hamilton Pleasure Scale; CTQ: Childhood Trauma Questionnaire; EN: emotional neglect; PN: physical neglect; EA: emotional abuse; PA: physical abuse; SA: sexual abuse; CT: childhood trauma.

**Table 2:** Comparison of anhedonia among MDD and OCD patients and university students, as well as between participants with and without CT in each sample.

	MDD		OCD		University students		MDD (*t*/*χ*^2^)			OCD (*t*/*χ*^2^)		University students (*t*/*χ*^2^)
	With CT (*N* = 213)	Without CT (*N* = 92)	With CT (*N* = 100)	Without CT (*N* = 52)	With CT (*N* = 709)	Without CT (*N* = 1401)	With vs. without CT	vs. university students	vs. OCD	With vs. without CT	vs. university students	With vs. without CT
TEPS-ANT	34.87 (9.28)	36.90 (9.53)	40.74 (8.57)	42.48 (6.90)	43.06 (8.06)	45.12 (8.06)	-1.18			-1.27		-5.55⁣^∗∗∗∗^
TEPS-CON	30.16 (7.92)	31.34 (8.20)	32.92 (7.45)	33.00 (7.25)	35.97 (7.21)	37.67 (7.49)	-1.74			-0.06		-4.99⁣^∗∗∗∗^
PAS	31.00 (10.83)	27.68 (11.1)	24.57 (10.55)	27.00 (12.3)	17.77 (7.78)	15.28 (7.32)	2.42⁣^∗^			-1.27		7.21⁣^∗∗∗∗^
SAS	22.17 (6.74)	19.23 (6.88)	17.87 (7.75)	18.83 (10.44)	12.78 (6.28)	10.21 (5.57)	3.46⁣^∗∗∗^			-0.64		9.59⁣^∗∗∗∗^
SHAPS^a^	34.32 (5.95)	32.10 (4.89)	32.57 (5.69)	30.95 (5.39)	25.45 (5.62)	23.36 (5.93)	1.79			1.09		7.76⁣^∗∗∗∗^
PAS (yes, %)	161 (75.6%)	60 (65.2%)	49 (49.0%)	25 (48.1%)	169 (23.8%)	198 (14.1%)	3.46	438.68⁣^∗∗∗∗^	25.06⁣^∗∗∗∗^	0.01	88.45⁣^∗∗∗∗^	30.85⁣^∗∗∗∗^
SAS (yes, %)	167 (78.4%)	59 (64.1%)	48 (48.0%)	24 (46.2%)	175 (24.7%)	147 (10.5%)	6.82⁣^∗∗^	525.88⁣^∗∗∗∗^	31.95⁣^∗∗∗∗^	0.05	101.62⁣^∗∗∗∗^	72.30⁣^∗∗∗∗^
SHAPS^a^ (yes, %)	63 (85.1%)	22 (75.9%)	33 (71.8%)	11 (52.4%)	192 (27.1%)	226 (16.1%)	1.24	219.92⁣^∗∗∗∗^	6.30⁣^∗^	2.40	81.70⁣^∗∗∗∗^	35.53⁣^∗∗∗∗^

^a^Sample sizes in MDD and OCD with SHAPS recorded were 103 (with CT = 74, without CT = 29) and 67 (with CT = 46, without CT = 21). ⁣^∗^*p* < 0.05, ⁣^∗∗^*p* < 0.01, ⁣^∗∗∗^*p* < 0.005, and ⁣^∗∗∗∗^*p* < 0.001. OCD: obsessive-compulsive disorder; MDD: major depression disorder; CT: childhood trauma; PAS: Physical Anhedonia Scale; SAS: Social Anhedonia Scale; SHAPS: Snaith-Hamilton Pleasure Scale.

**Table 3:** Correlations between anhedonia aspects and different types of childhood trauma in each sample.

	TEPS_ANT	TEPS_CON	PAS	SAS	SHAPS	BDI
*MDD*						
Neglect	**-0.18**⁣^∗∗∗^	**-0.17**⁣^∗∗∗^	**0.26**⁣^∗∗∗∗^	**0.25**⁣^∗∗∗∗^	**0.24**⁣^∗^	**0.24**⁣^∗∗∗∗^
Abuse	0.11	0.03	0.03	**0.16**⁣^∗∗^	0.04	**0.24**⁣^∗∗∗∗^
EN	**-0.17**⁣^∗∗∗^	**-0.17**⁣^∗∗∗^	**0.25**⁣^∗∗∗^	**0.22**⁣^∗∗∗∗^	**0.23**⁣^∗^	**0.21**⁣^∗∗∗∗^
PN	**-0.15**⁣^∗∗^	**-0.13**⁣^∗^	**0.20**⁣^∗∗∗∗^	**0.22**⁣^∗∗∗∗^	**0.20**⁣^∗^	**0.22**⁣^∗∗∗∗^
EA	0.09	0.04	0.07	**0.18**⁣^∗∗∗^	0.17	**0.25**⁣^∗∗∗∗^
PA	0.14	-0.03	-0.02	0.07	-0.10	0.10
SA	0.07	0.05	-0.03	0.10	-0.04	**0.17**⁣^∗∗∗^
CTQ_total	-0.05	-0.08	**0.17**⁣^∗∗∗^	**0.24**⁣^∗∗∗∗^	0.17	**0.28**⁣^∗∗∗∗^
*OCD*						
Neglect	**-0.24**⁣^∗∗∗^	-0.07	0.03	0.03	**0.37**⁣^∗∗∗^	**0.22**⁣^∗∗^
Abuse	0.06	0.08	0.02	0.04	0.16	**0.26**⁣^∗∗∗^
EN	**-0.25**⁣^∗∗∗^	-0.03	0.14	0.06	**0.32**⁣^∗∗^	**0.23**⁣^∗∗^
PN	-0.13	-0.10	-0.15	-0.03	**0.31**⁣^∗^	0.13
EA	0.05	0.10	0.01	0.02	0.22	**0.26**⁣^∗∗∗^
PA	0.04	0.05	0.10	0.07	0.03	0.14
SA	0.03	0.01	-0.08	<0.001	0.07	0.15
CTQ_total	-0.11	0.01	0.03	0.04	**0.33**⁣^∗∗^	**0.30**⁣^∗∗∗^
*University students*						
Neglect	**-0.20**⁣^∗∗∗∗^	**-0.17**⁣^∗∗∗∗^	**0.21**⁣^∗∗∗∗^	**0.26**⁣^∗∗∗∗^	**0.25**⁣^∗∗∗∗^	**0.30**⁣^∗∗∗∗^
Abuse	**-0.06**⁣^∗∗^	**-0.06**⁣^∗∗^	**0.11**⁣^∗∗∗∗^	**0.21**⁣^∗∗∗∗^	**0.15**⁣^∗∗∗∗^	**0.29**⁣^∗∗∗∗^
EN	**-0.21**⁣^∗∗∗∗^	**-0.16**⁣^∗∗∗∗^	**0.19**⁣^∗∗∗∗^	**0.27**⁣^∗∗∗∗^	**0.25**⁣^∗∗∗∗^	**0.30**⁣^∗∗∗∗^
PN	**-0.12**⁣^∗∗∗∗^	**-0.13**⁣^∗∗∗∗^	**0.16**⁣^∗∗∗∗^	**0.17**⁣^∗∗∗∗^	**0.18**⁣^∗∗∗∗^	**0.20**⁣^∗∗∗∗^
EA	-0.04	-0.04	**0.10**⁣^∗∗∗∗^	**0.22**⁣^∗∗∗∗^	**0.14**⁣^∗∗∗∗^	**0.29**⁣^∗∗∗∗^
PA	**-0.07**⁣^∗∗∗^	**-0.06**⁣^∗∗^	**0.09**⁣^∗∗∗∗^	**0.13**⁣^∗∗∗∗^	**0.09**⁣^∗∗∗∗^	**0.17**⁣^∗∗∗∗^
SA	-0.02	-0.03	**0.05**⁣^∗∗^	**0.11**⁣^∗∗∗∗^	**0.10**⁣^∗∗∗∗^	**0.17**⁣^∗∗∗∗^
CTQ_total	**-0.17**⁣^∗∗∗∗^	**-0.15**⁣^∗∗∗∗^	**0.20**⁣^∗∗∗∗^	**0.28**⁣^∗∗∗∗^	**0.25**⁣^∗∗∗∗^	**0.35**⁣^∗∗∗∗^

⁣^∗^*p* < 0.05, ⁣^∗∗^*p* < 0.01, ⁣^∗∗∗^*p* < 0.005, and ⁣^∗∗∗∗^*p* < 0.001. OCD: obsessive-compulsive disorder; MDD: major depression disorder; BDI: Beck Depression Inventory; TEPS_ANT: anticipatory pleasure subscale of Temporal Experience of Pleasure Scale; TEPS_CON: consummatory pleasure subscale of TEPS; PAS: Physical Anhedonia Scale; SAS: Social Anhedonia Scale; SHAPS: Snaith-Hamilton Pleasure Scale; CTQ: Childhood Trauma Questionnaire; EN: emotional neglect; PN: physical neglect; EA: emotional abuse; PA: physical abuse; SA: sexual abuse. Significant correlations were shown in bold values.

**Table 4:** Childhood neglect and abuse prediction of different aspects of anhedonia in MDD and OCD patients and university students.

Predictors	Variable statistics				Model statistics		
	*B* (s.e.)	*β*	*t*	*p*	Δ*R*^2^	*F*	*p*
MDD patients							
TEPS-ANT							
Step1: covariates					0.05	2.79	0.012
Step 2: neglect	**-0.18 (0.07)**	**-0.14**	**-2.48**	**0.014**	0.02	3.31	0.002
TEPS-CON							
Step 1: covariates					0.06	3.12	0.006
Step 2: neglect	**-0.15 (0.06)**	**-0.14**	**-2.40**	**0.017**	0.02	3.54	0.001
PAS							
Step 1: covariates					0.17	9.92	<0.001
Step 2: neglect	**0.28 (0.08)**	**0.19**	**3.59**	**<0.001**	0.03	10.67	<0.001
SAS							
Step 1: covariates					0.11	6.03	<0.001
Step 2: neglect	**0.17 (0.06)**	**0.18**	**2.84**	**0.005**	0.04	623	<0.001
Abuse	0.03 (0.06)	0.03	0.43	0.668			
SHAPS (*N* = 103)							
Step 1: covariates					0.17	3.36	0.005
Step 2: neglect	**0.19 (0.07)**	**0.25**	**2.65**	**0.009**	0.06	4.07	0.001
OCD patients							
TEPS-ANT							
Step 1: covariates					0.11	2.43	0.022
Step 2: neglect	**-0.26 (0.11)**	**-0.21**	**-2.47**	**0.015**	0.04	2.96	0.004
SHAPS (*N* = 67)							
Step 1: covariates					0.29	3.44	0.004
Step 2: neglect	**0.24 (0.10)**	**0.26**	**2.39**	**0.020**	0.06	3.96	0.001
University students							
TEPS-ANT							
Step1: covariates					0.09	35.63	<0.001
Step 2: neglect	**-0.23 (0.03)**	**-0.16**	**-6.83**	**<0.001**	0.02	33.31	0.001
Abuse	0.08 (0.05)	0.04	1.60	0.110			
TEPS-CON							
Step 1: covariates					0.07	26.71	<0.001
Step 2: neglect	**-0.16 (0.03)**	**-0.12**	**-5.50**	**<0.001**	0.01	23.40	<0.001
Abuse	0.07 (0.05)	0.04	1.49	0.137			
PAS							
Step 1: covariates					0.11	42.88	<0.001
Step 2: neglect	**0.14 (0.02)**	**0.10**	**4.35**	**<0.001**	0.01	35.32	<0.001
Abuse	0.01 (0.05)	0.003	0.11	0.909			
SAS							
Step 1: covariates					0.14	57.46	<0.001
Step 2: neglect	**0.12 (0.02)**	**0.11**	**4.81**	**<0.001**	0.03	52.03	<0.001
Abuse	**0.14 (0.04)**	**0.08**	**3.86**	**<0.001**			
SHAPS							
Step 1: covariates					0.14	54.83	<0.001
Step 2: neglect	**0.14 (0.02)**	**0.13**	**5.60**	**<0.001**	0.02	47.23	<0.001
Abuse	0.03 (0.04)	0.02	0.91	0.365			

OCD: obsessive-compulsive disorder; MDD: major depression disorder; TEPS_ANT: anticipatory pleasure subscale of Temporal Experience of Pleasure Scale; TEPS_CON: consummatory pleasure subscale of TEPS; PAS: Physical Anhedonia Scale; SAS: Social Anhedonia Scale; SHAPS: Snaith-Hamilton Pleasure Scale; CTQ: Childhood Trauma Questionnaire. Significant predictors were shown in bold values.

**Table 5:** Specific types of CT prediction of different aspects of anhedonia in MDD and OCD patients.

Predictors	Variable statistics				Model statistics		
	*B* (s.e.)	*β*	*t*	*p*	Δ*R*^2^	*F*	*p*
MDD patients							
TEPS-ANT							
Step 1: covariates					0.05	2.79	0.012
Step 2: EN	**-2.52 (0.11)**	**-0.14**	**-2.34**	**0.020**	0.02	3.21	0.003
PN (ex)			-0.80	0.425			
TEPS-CON							
Step 1: covariates					0.06	3.12	0.006
Step 2: EN	**-0.22 (0.09)**	**-0.14**	**-2.44**	**0.015**	0.02	3.57	0.001
PN (ex)			-0.36	0.718			
PAS							
Step 1: covariates					0.17	9.92	<0.001
Step 2: EN	**0.42 (0.12)**	**0.19**	**3.60**	**<0.001**	0.04	10.68	<0.001
PN (ex)			0.686	0.493			
SAS							
Step 1: covariates					0.11	6.03	<0.001
Step 2: PN	**0.36 (0.12)**	**0.18**	**3.14**	**0.002**	0.04	6.73	<0.001
EN (ex)			1.58	0.114			
EA (ex)			0.37	0.172			
SHAPS (*N* = 103)							
Step 1: covariates					0.17	3.36	0.005
Step 2: EN	**2.67 (0.11)**	**0.23**	**2.48**	**0.015**	0.05	3.92	0.001
PN (ex)			0.90	0.093			
OCD patients							
TEPS-ANT							
Step 1: covariates					0.11	2.43	0.022
Step 2: EN	**-0.40 (0.15)**	**-0.22**	**-2.67**	**0.008**	0.04	3.11	0.003
SHAPS (*N* = 67)							
Step 1: covariates					0.29	3.44	0.004
Step 2: PN	**0.55 (0.23)**	**0.27**	**2.44**	**0.018**	0.07	4.01	0.001
EN (ex)			0.75	0.455			

OCD: obsessive-compulsive disorder; MDD: major depression disorder; TEPS_ANT: anticipatory pleasure subscale of Temporal Experience of Pleasure Scale; TEPS_CON: consummatory pleasure subscale of TEPS; PAS: Physical Anhedonia Scale; SAS: Social Anhedonia Scale; SHAPS: Snaith-Hamilton Pleasure Scale; CT: childhood trauma; EN: emotional neglect; PN: physical neglect; ex: excluded from the regression model. Significant predictors were shown in bold values.

**Table 6:** Specific types of CT prediction of different aspects of anhedonia in university students.

Predictors	Variable statistics				Model statistics		
	*B* (s.e.)	*β*	*t*	*p*	Δ*R*^2^	*F*	*p*
University students							
TEPS-ANT							
Step 1: covariates					0.09	35.63	<0.001
Step 2: EN	**-0.36 (0.05)**	**-0.17**	**-7.74**	**<0.001**	0.03	39.97	<0.001
PN (ex)			0.86	0.770			
PA (ex)			0.86	0.390			
TEPS-CON							
Step 1: covariates					0.07	26.71	<0.001
Step 2: EN	**-0.20 (0.04)**	**-0.10**	**-4.61**	**<0.001**	0.01	26.16	<0.001
PN (ex)			-1.49	0.136			
PA (ex)			0.41	0.679			
PAS							
Step 1: covariates					0.11	42.88	<0.001
Step 2: EN	**0.20 (0.04)**	**0.10**	**4.66**	**<0.001**	0.01	40.22	<0.001
PN (ex)			1.37	0.172			
PA (ex)			0.79	0.428			
EA (ex)			-0.03	0.973			
SA (ex)			-0.59	0.553			
SAS							
Step 1: covariates					0.14	57.46	<0.001
Step 2: EN	**0.20 (0.04)**	**0.13**	**5.67**	**<0.001**	0.03	53.68	<0.001
EA	**0.27 (0.07)**	**0.09**	**4.10**	**<0.001**			
PA (ex)			0.13	0.897			
PN (ex)			0.39	0.698			
SA (ex)			0.37	0.714			
SHAPS							
Step 1: covariates					0.14	54.83	<0.001
Step 2: EN	**0.22 (0.03)**	**0.14**	**6.54**	**<0.001**	0.02	54.03	<0.001
PN (ex)			1.40	0.161			
PA (ex)			-0.01	0.990			
EA (ex)			0.99	0.322			
SA (ex)			0.75	0.452			

TEPS_ANT: anticipatory pleasure subscale of Temporal Experience of Pleasure Scale; TEPS_CON: consummatory pleasure subscale of TEPS; PAS: Physical Anhedonia Scale; SAS: Social Anhedonia Scale; SHAPS: Snaith-Hamilton Pleasure Scale; CT: childhood trauma; EN: emotional neglect; PN: physical neglect; EA: emotional abuse; PA: physical abuse; SA: sexual abuse; ex: excluded from the regression model. Significant predictors were shown in bold values.

## Data Availability

The data that support the findings of this study are available from the corresponding author upon reasonable request.

## Supplementary Materials

Supplementary Materials Table S1: prediction of variables in step 1 for regression models in MDD. Table S2: prediction of variables in step 1 for regression models in OCD. Table S3: prediction of variables in step 1 for regression models in university students.
