# Supplementary material for: Childhood Neglect rather than Abuse Is More Strongly Associated with Anhedonia across Major Depression and Obsessive-Compulsive Disorder Patients and University Students
Source: Depress Anxiety. 2023 Oct 20;2023:2429889. doi: 10.1155/2023/2429889 (PMC11921836; doi:10.1155/2023/2429889)
Supplement: Supplementary Materials — Table S1: prediction of variables in step 1 for regression models in MDD. Table S2: prediction of variables in step 1 for regression models in OCD. Table S3: prediction of variables in step 1 for regression models in university students. [file 2429889.f1.docx]

**SUPPLEMENTARY MATERIALS**

**Table S1** Prediction of variables in step 1 for regression models in MDD.

| **Predictors** | **Variable statistics** | | | | | **Model statistics** | | |
| --- | --- | --- | --- | --- | --- | --- | --- | --- |
|  | ***B (s.e.)*** | ***β*** | ***t*** | ***p*** |  | ***ΔR^2^*** | ***F*** | ***p*** |
| ***TEPS-ANT*** |  |  |  |  | |  |  |  |
| Step 1 (for all models) |  |  |  |  | | 0.05 | 2.79 | 0.012 |
| Age | 0.01(0.10) | 0.003 | 0.06 | 0.953 | |  |  |  |
| Gender | 1.63(1.18) | 0.08 | 1.38 | 0.169 | |  |  |  |
| Education years | 0.25(0.23) | 0.07 | 1.11 | 0.267 | |  |  |  |
| SAI | -0.19(0.07) | -0.21 | -2.66 | 0.011 | |  |  |  |
| TAI | 0.10(0.11) | 0.081 | 0.901 | 0.368 | |  |  |  |
| PSS | -0.05(0.13) | -0.03 | -0.35 | 0.709 | |  |  |  |
| ***TEPS-CON*** |  |  |  |  | |  |  |  |
| Step 1 (for all models) |  |  |  |  | | 0.06 | 3.12 | 0.006 |
| Age | -0.04(0.08) | -0.03 | -0.47 | 0.637 | |  |  |  |
| Gender | 0.70(1.00) | 0.04 | 0.69 | 0.448 | |  |  |  |
| Education years | 0.54(0.19) | 0.17 | 2.81 | 0.005 | |  |  |  |
| SAI | -0.02(0.06) | -0.02 | -0.27 | 0.791 | |  |  |  |
| TAI | 0.03(0.09) | 0.029 | 0.33 | 0.742 | |  |  |  |
| PSS | -0.22(0.11) | -0.15 | -2.06 | 0.040 | |  |  |  |
| ***PAS*** |  |  |  |  | |  |  |  |
| Step 1 (for all models) |  |  |  |  | | 0.17 | 9.92 | <0.001 |
| Age | 0.06(0.11) | 0.03 | 0.60 | 0.551 | |  |  |  |
| Gender | -0.01(1.28) | <0.001 | -0.01 | 0.994 | |  |  |  |
| Education years | -0.68(0.25) | -0.15 | -2.76 | 0.006 | |  |  |  |
| SAI | 0.17(0.08) | 0.16 | 2.14 | 0.033 | |  |  |  |
| TAI | 0.03(0.12) | 0.02 | 0.28 | 0.782 | |  |  |  |
| PSS | 0.39(0.14) | 0.19 | 2.76 | 0.006 | |  |  |  |
| ***SAS*** |  |  |  |  | |  |  |  |
| Step 1 (for all models) |  |  |  |  | | 0.11 | 6.03 | <0.001 |
| Age | -0.09(0.07) | -0.07 | -1.25 | 0.213 | |  |  |  |
| Gender | -1.31(0.83) | -0.09 | -1.57 | 0.118 | |  |  |  |
| Education years | -0.15(0.16) | -0.05 | -0.90 | 0.367 | |  |  |  |
| SAI | 0.15(0.05) | 0.23 | 2.93 | 0.004 | |  |  |  |
| TAI | -0.05(0.08) | -0.05 | -0.58 | 0.560 | |  |  |  |
| PSS | 0.17(0.09) | 0.14 | 1.86 | 0.064 | |  |  |  |
| ***SHAPS (N = 103)*** |  |  |  |  | |  |  |  |
| Step 1 (for all models) |  |  |  |  | | 0.17 | 3.36 | 0.005 |
| Age | -0.07(0.18) | -0.04 | -0.37 | 0.711 | |  |  |  |
| Gender | -0.46(1.23) | -0.04 | -0.38 | 0.708 | |  |  |  |
| Education years | -0.12(0.25) | -0.06 | -0.47 | 0.637 | |  |  |  |
| SAI | 0.04(0.07) | 0.07 | 0.58 | 0.565 | |  |  |  |
| TAI | 0.24(0.11) | 0.28 | 2.08 | 0.040 | |  |  |  |
| PSS | 0.14(0.12) | 0.14 | 1.15 | 0.251 | |  |  |  |

MDD, Major depression disorder; TEPS_ANT, anticipatory pleasure subscale of Temporal Experience of Pleasure Scale; TEPS_CON, consummatory pleasure subscale of TEPS; PAS, Physical Anhedonia Scale; SAS, Social Anhedonia Scale; SHAPS, Snaith-Hamilton Pleasure Scale; SAI, State Anxiety Inventory; TAI, Trait Anxiety Inventory; PSS, Perceived Stress Scale.

**Table S2** Prediction of variables in step 1 for regression models in OCD.

| **Predictors** | **Variable statistics** | | | | | **Model statistics** | | |
| --- | --- | --- | --- | --- | --- | --- | --- | --- |
|  | ***B (s.e.)*** | ***β*** | ***t*** | ***p*** |  | ***ΔR^2^*** | ***F*** | ***p*** |
| ***TEPS-ANT*** |  |  |  |  | |  |  |  |
| Step 1 (for all models) |  |  |  |  | | 0.11 | 2.43 | 0.022 |
| Age | 0.08(0.13) | 0.06 | 0.64 | 0.520 | |  |  |  |
| Gender | -0.26(1.32) | -0.16 | -0.20 | 0.845 | |  |  |  |
| Education years | -0.09(0.26) | -0.03 | -0.36 | 0.723 | |  |  |  |
| SAI | 0.20(0.12) | 0.16 | 1.70 | 0.092 | |  |  |  |
| TAI | 0.05(0.14) | 0.04 | 0.35 | 0.722 | |  |  |  |
| PSS | 0.21(0.18) | 0.11 | 1.15 | 0.251 | |  |  |  |
| Y-BOCS | -0.29(0.10) | -0.22 | -2.78 | 0.006 | |  |  |  |
| ***SHAPS (N = 67)*** |  |  |  |  | |  |  |  |
| Step 1 (for all models) |  |  |  |  | | 0.29 | 3.44 | 0.004 |
| Age | -0.02(0.16) | -0.01 | -0.10 | 0.924 | |  |  |  |
| Gender | 1.54(1.30) | 0.14 | 1.19 | 0.240 | |  |  |  |
| Education years | -0.09(0.29) | -0.04 | -0.31 | 0.759 | |  |  |  |
| SAI | -0.34(0.12) | -0.38 | -2.81 | 0.007 | |  |  |  |
| TAI | -0.12(0.17) | -0.12 | -0.71 | 0.483 | |  |  |  |
| PSS | 0.50(0.19) | 0.21 | 1.80 | 0.078 | |  |  |  |
| Y-BOCS | 0.18(0.10) | 0.21 | 1.80 | 0.078 | |  |  |  |

OCD, obsessive-compulsive disorder; TEPS_ANT, anticipatory pleasure subscale of Temporal Experience of Pleasure Scale; SHAPS, Snaith-Hamilton Pleasure Scale; SAI, State Anxiety Inventory; TAI, Trait Anxiety Inventory; PSS, Perceived Stress Scale; Y-BOCS, Yale-Brown obsessive compulsive scale.

**Table S3** Prediction of variables in step 1 for regression models in university students.

| **Predictors** | **Variable statistics** | | | | | **Model statistics** | | |
| --- | --- | --- | --- | --- | --- | --- | --- | --- |
|  | ***B (s.e.)*** | ***β*** | ***t*** | ***p*** |  | ***ΔR^2^*** | ***F*** | ***p*** |
| ***TEPS-ANT*** |  |  |  |  | |  |  |  |
| Step 1 (for all models) |  |  |  |  | | 0.09 | 35.63 | <0.001 |
| Age | -0.48(0.17) | -0.08 | -2.86 | 0.004 | |  |  |  |
| Gender | 3.70(0.34) | 0.23 | 10.93 | <0.001 | |  |  |  |
| Education years | 0.48(0.21) | 0.06 | 2.30 | 0.022 | |  |  |  |
| SAI | -0.07(0.03) | -0.08 | -2.04 | 0.041 | |  |  |  |
| TAI | -0.10(0.04) | -0.12 | -2.74 | 0.006 | |  |  |  |
| PSS | 0.17(0.04) | 0.12 | 4.22 | <0.001 | |  |  |  |
| ***TEPS-CON*** |  |  |  |  | |  |  |  |
| Step 1 (for all models) |  |  |  |  | | 0.07 | 26.71 | <0.001 |
| Age | -0.41(0.16) | -0.07 | -2.65 | 0.008 | |  |  |  |
| Gender | 2.50(0.32) | 0.17 | 7.92 | <0.001 | |  |  |  |
| Education years | 0.39(0.20) | 0.05 | 1.97 | 0.049 | |  |  |  |
| SAI | 0.001(0.03) | 0.001 | 0.02 | 0.984 | |  |  |  |
| TAI | -0.16(0.03) | -0.21 | -4.71 | <0.001 | |  |  |  |
| PSS | 0.08(0.04) | 0.06 | 2.10 | 0.036 | |  |  |  |
| ***PAS*** |  |  |  |  | |  |  |  |
| Step 1 (for all models) |  |  |  |  | | 0.11 | 42.88 | <0.001 |
| Age | -0.40(0.20) | -0.05 | -1.95 | 0.052 | |  |  |  |
| Gender | -2.16(0.32) | -0.14 | -6.85 | <0.001 | |  |  |  |
| Education years | -0.38(0.20) | -0.05 | -1.95 | 0.052 | |  |  |  |
| SAI | 0.03(0.03) | 0.04 | 0.994 | 0.320 | |  |  |  |
| TAI | 0.15(0.03) | 0.19 | 4.45 | <0.001 | |  |  |  |
| PSS | 0.06(0.04) | 1.65 | 1.65 | 0.100 | |  |  |  |
| ***SAS*** |  |  |  |  | |  |  |  |
| Step 1 (for all models) |  |  |  |  | | 0.14 | 57.46 | <0.001 |
| Age | 0.02(0.12) | 0.004 | 0.17 | 0.866 | |  |  |  |
| Gender | -0.61(0.24) | -0.05 | -2.52 | 0.012 | |  |  |  |
| Education years | -0.08(0.15) | -0.01 | -0.52 | 0.602 | |  |  |  |
| SAI | 0.02(0.02) | 0.03 | 0.75 | 0.451 | |  |  |  |
| TAI | 0.14(0.03) | 0.23 | 5.48 | <0.001 | |  |  |  |
| PSS | 0.08(0.03) | 0.08 | 2.73 | 0.006 | |  |  |  |
| ***SHAPS*** |  |  |  |  | |  |  |  |
| Step 1 (for all models) |  |  |  |  | | 0.14 | 54.83 | <0.001 |
| Age | 0.07(0.12) | 0.01 | 0.57 | 0.570 | |  |  |  |
| Gender | -1.46(0.24) | -0.12 | -6.07 | <0.001 | |  |  |  |
| Education years | -0.13(0.02) | -0.02 | -0.84 | 0.402 | |  |  |  |
| SAI | 0.02(0.02) | 0.04 | 0.89 | 0.375 | |  |  |  |
| TAI | 0.16(0.03) | 0.26 | 6.16 | <0.001 | |  |  |  |
| PSS | 0.01(0.03) | 0.01 | 0.48 | 0.631 | |  |  |  |

TEPS_ANT, anticipatory pleasure subscale of Temporal Experience of Pleasure Scale; TEPS_CON, consummatory pleasure subscale of TEPS; PAS, Physical Anhedonia Scale; SAS, Social Anhedonia Scale; SHAPS, Snaith-Hamilton Pleasure Scale; SAI, State Anxiety Inventory; TAI, Trait Anxiety Inventory; PSS, Perceived Stress Scale.
